# Emphysematous cystitis in a patient using immunosuppressant agents

**DOI:** 10.1002/jgf2.399

**Published:** 2020-11-07

**Authors:** Daiki Yokokawa, Takanori Uehara, Masatomi Ikusaka

**Affiliations:** ^1^ Department of General Medicine Chiba University Hospital Chiba Japan

**Keywords:** emphysematous cystitis, immunosuppressant, infectious diseases, internal medicine, rheumatoid arthritis, urology

## Abstract

A 72‐year‐old woman presented with a 3‐day history of nausea, vomiting, and fever. She had rheumatoid arthritis and was taking prednisolone (10 mg), cyclosporine (150 mg), and actarit (200 mg) daily. Computed tomography revealed gases were detected in the bladder wall, and emphysematous cystitis was diagnosed. When an immunocompromised host is suspected of a severe urinary tract infection but lacks specific signs or symptoms such as costovertebral angle tapping pain, emphysematous cystitis should be considered.

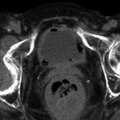

A 72‐year‐old woman presented with a 3‐day history of nausea, vomiting, and fever. She had rheumatoid arthritis and was taking prednisolone (10 mg), cyclosporine (150 mg), and actarit (200 mg) daily. She denied dysuria, frequent urination, or pain on miction. Her temperature was 37.5°C, but other vital signs were normal. She had no costovertebral angle (CVA) tapping pain. Urinalysis showed pyuria/microscopic hematuria. Laboratory tests showed leukocytosis. Computed tomography revealed gases were detected in the bladder wall (Figure 1), and emphysematous cystitis (EC) was diagnosed. Meropenem was started for empiric therapy. A Gram stain of urine revealed Gram‐negative bacilli. A urine culture indicated the presence of *Escherichia coli*, and anaerobic blood culture indicated the presence of *Parvimonas micra*. We continued antibiotic therapy, and her general condition improved.

**FIGURE 1:**
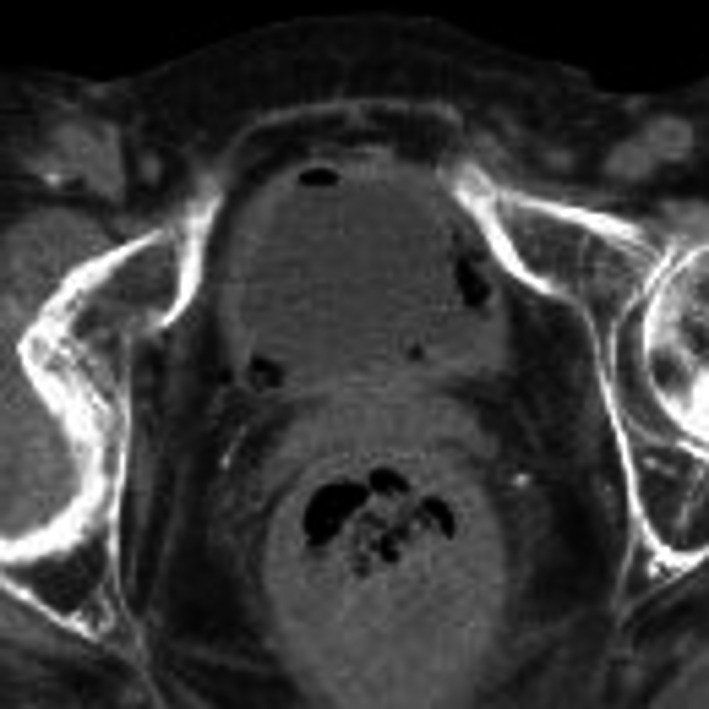
A computed tomography scan showing gases within the bladder and bladder wall

EC is a rare disease caused by gas‐forming bacteria included *Escherichia coli* and *Klebsiella pneumoniae*. It is common in immunocompromised patients, and a lethal course has been reported in 7% of cases.^1^ Symptoms are nonspecific and unclear.^2^ In this case, *Parvimonas micra* detected in the two anaerobic blood culture bottles; however, an association with EC has not been previously reported. When an immunocompromised host is suspected of a severe urinary tract infection but lacks specific signs or symptoms such as CVA tapping pain, EC should be considered.

## CONFLICT OF INTEREST

The authors declare that they do not have a conflict of interest.

## AUTHOR CONTRIBUTIONS

All authors had access to the data and a role in writing the manuscript.

## INFORMED CONSENT STATEMENT

Informed written consent was obtained from the patient for publication of this report and any accompanying images.
